# Short-term clinical outcomes of 220 dogs with thoraco-lumbar disc disease treated by mini-hemilaminectomy

**DOI:** 10.4102/jsava.v91i0.2008

**Published:** 2020-12-21

**Authors:** Ross C. Elliott, Chantel Moon, Gareth Zeiler, Remo Lobetti

**Affiliations:** 1 Department of Companion Animal Clinical Studies, University of Pretoria, Onderstepoort, South Africa; 2 Department of Small Animal Surgery, Bryanston Veterinary Hospital, Bryanston, South Africa; 3 Department of Small Animal Medicine, Bryanston Veterinary Hospital, Bryanston, South Africa

**Keywords:** spinal, surgery, small animals, mini-hemilaminectomy, disc extrusion

## Abstract

Thoraco-lumbar intervertebral disc extrusion is a common condition seen in veterinary practice. Although there are different surgical techniques described for decompression, most of these techniques are based on the surgeon’s preference or experience rather than clinical research. Our objective was to determine the clinical outcomes, using return to ambulation and micturition, as well as complication rates, in a large cohort of dogs by using a mini-hemilaminectomy for decompression of the thoraco-lumbar spinal cord with Hansen type I thoraco-lumbar intervertebral disc extrusions (IVDE). A retrospective study was performed on dogs presented for acute thoraco-lumbar IVDE undergoing surgical decompression. In total, 252 spinal decompression surgeries were performed. The recovery rates for patients graded with a modified Frankel score (MFS) of 5 to 0 were 100%, 99%, 100%, 96%, 86% and 64%, respectively. The mean days to micturition across all the MFS 5–0 were 1.5 (standard deviation [SD] ± 0.7), 1.8 (SD ± 1), 4.3 (SD ± 1.7), 6.4 (SD ± 2.2), 9.3 (SD 3) and 11.9 (SD ± 2.2), respectively. The mean days to ambulation across all the groups 5–0 were 2 (SD ± 0.7), 2.6 (SD ± 1), 7.6 (SD ± 4.4), 10.1 (SD ± 2.5), 16.1 (SD ± 2.9) and 19.3 (SD ± 2.6), respectively. Postoperative complications were seen in 32 of the surgeries, with a complication rate of 13%. Minor complications accounted for 38% of all complications, and major complications constituted 62% of all complications. In total, 15 dogs died or were euthanised as a direct result of thoraco-lumbar disc extrusion or the surgical procedure, with a mortality rate of 6% across all groups. A mini-hemilaminectomy provides similar clinical outcomes described in the literature for other methods of spinal cord decompressive surgery, and it also provides patients with similar short-term outcomes to other described decompressive surgical techniques in the dog, which have been described in the literature.

## Introduction

Canine thoraco-lumbar intervertebral disc extrusion (IVDE) is a common condition presenting to the small animal veterinarian. The condition is most commonly seen in chondrodystrophic dogs but has been reported in all breeds (Sharp & Wheeler 2005). The disease has a peak incidence in patients aged 3–6 years (Sharp & Wheeler 2005). The disease has clinical signs that are usually acute in onset, with the most common clinical signs being thoraco-lumbar paraspinal hyperaesthesia, paraparesis to paraplegia and loss of nociception.

Patients are generally graded in regard to the degree of neurological dysfunction (Griffin, Levine & Kerwin 2009; Levine et al. 2009; Olby et al. 2001), allowing prognostication for both the owner and veterinarian. The most important prognostic indicator in IVDE is pelvic limb deep pain sensation (Griffin et al. 2009; Levine et al. 2009; Olby et al. 2001). There have been multiple grading systems introduced over the past few years, but the modified Frankel score (MFS) (Table 1) is the most commonly used (Griffin et al. 2009; Levine & Caywood 1984). The presence of ‘deep pain’ is tested by delivering a noxious stimulus to one of the pelvic limb digits and requires the animal to have a central reaction to the stimulus such as vocalisation or turning to acknowledge the stimulus. The MFS has shown to prognosticate the recovery in dogs with IVDE (Davis & Brown 2002; Griffin et al. 2009; Ingram, Kale & Balfour 2013; Levine et al. 2009; Skytte & Schmökel 2018). Dogs with an MFS of 5–2 at presentation have a success rate of 97% – 95% with decompressive surgery (Ingram et al. 2013; Olby et al. 2003; Skytte & Schmökel 2018; Sukhiani, Parent & Atilola 1996). Dogs with no deep pain response (MFS 0) if operated on within the first 24 h of losing deep pain sensation have a 64% chance of having a successful outcome (Ingram et al. 2013; Scott & McKee 1999; Skytte & Schmökel 2018).

**TABLE 1:** The modified Frankel score used to prognosticate in spinal cord injury in dogs.

Grade	Description
5	Normal gait with paraspinal hyperaesthesia
4	Ambulatory para/tetraparesis (ataxia)
3a	Non-ambulatory para/tetraparesis with the ability to bear weight on the limbs without support
3b	Non-ambulatory para/tetraparesis with the inability to bear weight on the limbs without support
2	Para/tetraplegia with intact nociception
1	Para/tetraplegia with loss of superficial nociception
0	Para/tetraplegia with loss of deep nociception

*Source*: Levine, G.J., Levine, J.M., Budke, C.M., Kerwin, S.C., Au, J., Vinayak, A. et al., 2009, ‘Description and repeatability of a newly developed spinal cord injury scale for dogs’, *Preventive Veterinary Medicine* 89(1–2), 121–127. https://doi.org/10.1016/j.prevetmed.2009.02.016

Prior to performing surgery, the diagnosis and the location of surgical decompression need to be confirmed. This should always be performed by using advanced imaging studies such as myelography, computed tomography or magnetic resonance imaging (Besalti et al. 2006; Bos et al. 2012; Burk 1989; De Decker et al. 2016; Newcomb et al. 2012).

Although there are no strict guidelines on when surgery should be performed over medical management, non-ambulatory and ambulatory dogs with paraspinal hyperaesthesia that are non-responsive to medical management are most often treated surgically (Langerhuus & Miles 2017; McKee 1992; Scott 1997; eds. Sharp & Wheeler 2005). The recovery rate with surgical decompression of the spine is more rapid than with fenestration or non-surgical treatment according to some publications (Ferreira, Correia & Jaggy 2002; Langerhuus & Miles 2017; McKee 1992; Rousse et al. 2016). The most commonly used surgical decompressive technique for an IVDE of the thoraco-lumbar spinal cord in canines is a hemilaminectomy (Ferreira et al. 2002; McKee 1992). A hemilaminectomy involves removal of the articular facet joint, the caudal vertebral pedicle of the cranial vertebra and the cranial pedicle of the caudal vertebra. This technique provides excellent access to the extruded disc material and the disc for fenestration compared with a dorsal laminectomy whilst limiting iatrogenic damage to the nerve root (McKee 1992). A mini-hemilaminectomy (Images 1 and 2), however, preserves the articular facet joint and only removes the pedicle, described as for a hemilaminectomy ventral to the articular facet (Lubbe, Kirberger & Verstraele 1994). Studies have observed residual spinal cord compression post-operatively when a mini-hemilaminectomy was compared with a hemilaminectomy (Huska et al. 2014; Svensson et al. 2017). A recent study has however contraindicated this by indicating less residual disc material with a mini-hemilaminectomy compared with a hemilaminectomy (Svensson et al. 2017). Hemilaminectomy has however been shown in mechanical studies to increase the range of motion of the vertebral column by 11%; this has been put forward as a cause of instability in dogs recovering from decompressive surgery of the spinal cord (Hill, Lubbe & Guthrie 2000). Studies have failed to indicate significant differences in stability when comparing a hemilaminectomy with a mini-hemilaminectomy (Arthurs 2009; De Vicente et al. 2013). The access to the vertebral canal provided by a mini-hemilaminectomy has been assumed to be less than that of a hemilaminectomy, but to the authors’ knowledge the difference in clinical outcomes has not been reported.

Hospitalisation of patients can be prolonged in IVDE with a low MFS. Patients are often sent home once micturition and early ambulation have returned (MFS 3–4). These factors are often used as an end point in studies conducted to determine the success of decompressive surgery (Aikawa et al. 2012; Atalan et al. 2002; Skytte & Schmökel 2018).

Surgical complications are an unfortunate reality of all surgical procedures. These complications are classified as major complications and minor complications. Major complications are catastrophic to the well-being of the patient or require a repeat surgical procedure. Minor complications are complications that do not require a repeated surgical intervention and can be managed with conservative treatment, and there is no effect on the length of hospitalisation or clinical outcome.

Common complications related to the surgical procedure generally occur in a small number of patients undergoing spinal cord decompression surgery (Ingram et al. 2013; Scott 1997; Skytte & Schmökel 2018). Common intraoperative complications that have been reported include improper identification of the surgical site, residual disc material at the surgical site post-decompression, excessive haemorrhage from the venous sinuses and iatrogenic injury to the spinal cord (Bruniges & Rioja 2019; Dhupa et al. 1999a, 1999b; Sharp & Wheeler 2005). Hypothermia, hypotension and regurgitation are the most commonly seen anaesthetic complications associated with hemilaminectomy (Bruniges & Rioja 2019; Stiffler et al. 2006).

Common complications seen in the immediate post-operative period that are directly related to surgical decompression of the spinal cord will affect a small number of patients. These include seroma formation at the surgical site, surgical site infection, repeat IVDE at the operated surgical site or another disc space, aseptic necrosis of the fat graft, incontinence, no recovery of neurological function and ascending or descending myelomalacia (Aikawa et al. 2012; Balducci et al. 2017; Castel et al. 2017; Dyall & Schmökel 2018; Olby et al. 2003; Rousse et al. 2016; Scott 1997).

Patients with IVDE may also be affected by other complications in the immediate post-operative recovery period. These complications are not directly related to the decompressive surgery; however, they are related to the effects of the spinal cord damage that has occurred. The most common complications are urinary tract infections; pressure sores; post-operative pain; self-mutilation of the pelvic limbs, penis and tail; and bronchopneumonia (Ingram et al. 2013; Java et al. 2009; Sharp & Wheeler 2005).

The objective of this study was to evaluate the surgical outcomes of a large cohort of dogs treated with a mini-hemilaminectomy for acute IVDE.

## Materials and methods

Hospital records of all dogs admitted to the Bryanston Veterinary Hospital between January 2012 and December 2014 were searched. Dogs weighing < 15 kg presenting with an acute onset of signs relating to a thoraco-lumbar myelopathy less than 7 days of duration were included in the study. These records were examined for cases that had an acute Hansen type I IVDE and that underwent decompressive surgery via a mini-hemilaminectomy. Data from these records were retrieved by a co-author (C.M.). The patient’s age, weight, breed, preoperative MFS (Table 1), site of spinal cord compression, number of days till return of normal micturition, number of days till return of ambulation (MFS 4), minor and major complications attributed to the decompressive surgery or the disc extrusion itself were recorded. The MFS was used to grade dogs into groups based on their neurological status before surgery to assign them a 6-point scale of 5–0, thus assigning them to a specific group. In the MFS grade system, grade 3 is split into two subsections A and B; for the purpose of this publication, all dogs in these two subsections were grouped together as grade 3.

Normal micturition was determined by active urination by the dog after which the size of the bladder was palpated or abdominal ultrasound was performed to ensure complete emptying of the bladder. This was based on the percentage of bladder emptying as reported in the study by Atalan et al. 2002.

The dogs’ clinical records were inspected retrospectively. All the information in the clinical records was recorded in most cases by the primary author (R.E.), a registered surgical specialist or a surgical resident.

Dogs with incomplete clinical records were excluded from the study, and dogs that died because of reasons unrelated to the IVDE were removed from the study. Also, dogs that died or were euthanised because of complications arising from the decompressive surgery or the IVDE were not included in the analysis for days to micturition and ambulation, but they were included in the complications and recovery rate of their specific group.

A recovery in this study was defined as a dog with return of normal micturition and the ability to ambulate without injuring itself (MFS 4).

This study assessed the clinical outcomes of mean days to return of ambulation and normal micturition as this represented a recovery. Dogs that never regained ambulation or normal micturition were classified as unsuccessful outcomes. These dogs were removed from the statistical analysis for days to micturition and ambulation and classified as dogs with major complications in their respective groups.

The numbers of minor and major complications were recorded per group. In cases of a suspected surgical site infection, the surgical site was aseptically prepared, a needle aspirate was taken from the surgical site and submitted for aerobic bacterial culture and antibiogram. This differentiated a seroma from a surgical site infection. Any dog showing clinical signs of stranguria, dysuria or haematuria had a cystocentesis performed and the urine sample was sent for aerobic bacterial culture and antibiogram to diagnose a urinary tract infection.

Any patient that died postoperatively in the hospital was submitted for a post-mortem to an independent pathologist.

### Diagnostic imaging

All dogs presenting with IVDE were pre-medicated with diazepam (0.2 mg/kg IV, Valium, Roche products, Illovo, Johannesburg) and morphine (0.5 mg/kg IV Pharma-Q-Morphine, Pharma-Q Holdings, Industria West, Johannesburg). Induction was performed with propofol (6.6 mg/kg IV, Fresenius Propoven, Fresenius Kabi, Midrand, Johannesburg). Patients were intubated and maintained on isoflurane (Isofor 100 mL, Safeline pharmaceuticals, Roodepoort, Johannesburg) in 100% oxygen.

Right lateral, left lateral and ventro-dorsal survey radiographs were taken. An area over the dorsal lumbar spine was clipped and surgically prepared for lumbar myelogram, which was performed by injecting Iohexal (Ominpaque, 0.3 mL/kg, 140 mg/mL, GE Healthcare, Midrand, Johannesburg) into the sub-arachnoid space. Ventro-dorsal, left lateral, left ventro-dorsal oblique and right ventro-dorsal oblique radiographs were taken to complete the myelographic study.

### Surgical technique

The entire dorsal aspect of the thoraco-lumbar spine was then clipped and aseptically prepared for surgical decompression. All operations were performed by the same specialist surgeon (R.E.). A mini-hemilaminectomy (Lubbe et al. 1994) was performed by using a pneumatic burr (Pen-drive^TM^ Synthes). The disc material was visualised and removed. The disc was fenestrated, the area lavaged with warm ringers lactate and a thin free fat graft placed over the mini-hemilaminectomy site.

The urinary bladder was expressed during the recovery from anaesthesia. If this was not possible, the dog was catheterised to ensure an empty bladder for an accurate assessment of early return of bladder function.

Post-operatively, dogs were maintained on intravenous fluid therapy and morphine (0.5 mg/kg IV Pharma-Q-Morphine, Pharma-Q Holdings, Industria West, Johannesburg) over a 24-h period. Antibiotic cover with cephalosporin (20 mg/kg Zefkol IV Aspen, Claremont, Cape town) was provided throughout the night. A fentanyl patch (durogesic 25 *μ*g transdermal, Janssen, Woodmead, Johannesburg) was placed on completion of surgery to maintain analgesia in the 72 h post-surgery.

### Statistical analysis

Using Prism 8 software (GraphPad Software, San Diego, United States), correlations between intervals to regaining micturition and ambulation and minor and major complications were calculated by conducting the Spearman test. Values of *p* < 0.05 were considered to be significant.

### Ethical consideration

Ethical clearance was not required for the study. All work was carried out on clinical cases treated with the current standard of care recommended in veterinary medicine. All data were gathered from clinical cases treated at the Bryanston Veterinary Hospital.

## Results

All subjects in this study were canines with an age range of 3–12 years. Dachshunds accounted for 171 (68%) of the 252 patients and were the most commonly presented breed for decompressive surgery for thoraco-lumbar disc extrusions.

In total, 252 spinal decompressive surgeries were performed (Table 2). Of these dogs that were presented for decompressive surgery, 192 dogs had a single decompressive surgery in the 2-year period and 35 dogs had two or more decompressive surgeries at separate sites. Of these 35 dogs with repeat decompressive surgeries, 30 dogs had undergone two surgeries and 5 had three decompressive surgeries in the 2-year period (Table 2).

**TABLE 2:** Patients grouped by using the modified Frankel score.

Modified Frankel Score Group	Number of mini-hemilaminectomies	Number of dachshunds		Time to micturition days		Time to ambulation days		Minor complications number		Major complications numbers		Patients euthanised	Patients managed with wheels	Patients died	Patients recovered	
		*N*	%	Mean	SD	Mean	SD	*N*	%	*N*	%				*N*	%
5	14	10	71	1.5	0.7	2	0.7	0	0	1	7	0	0	0	14	100
4	99	65	66	1.8	1	2.6	1	2	2	1	1	1	0	0	98	99
3a and b	33	26	78	4.3	1.7	7.6	4.4	0	0	0	0	0	0	0	33	100
2	49	32	65	6.4	2.2	10.1	2.5	2	4	3	6	2	0	0	47	96
1	29	18	62	9.3	3	16.1	2.9	5	17	4	14	3	0	1	25	86
0	28	20	71	11.9	2.2	19.3	2.6	4	14	10	36	5	2	3	19	64
*p*	-	0.65	-	0.004	-	0.004	-	0.04	-	0.03	-	-	-	-	-	-

Note: The number of dogs, breed of dogs, mean time to ambulation, mean time to micturition, major and minor complications, and the recovery for each group are shown.

Correlations between intervals to regaining micturition and ambulation and minor and major complications were calculated by using the Spearman test. Values of *p* < 0.05 were considered to be significant.

A significant (*p* < 0.05) negative correlation (*r* = –0.68) was observed between preoperative MFS and interval to regaining micturition.

A significant (*p* < 0.05) negative (*r* = –0.59) correlation was identified between preoperative MFS and interval to regaining ambulation.

A significant (*p* < 0.05) negative (*r* = –0.61 and –0.67) correlation was observed between preoperative MFS and development of minor and major complications, respectively.

In total, 236 dogs recovered from mini-hemilaminectomy across all the groups. The majority of dogs (99 dogs) presented with an MFS of 4. The lowest number of dogs (14 dogs) presented with an MFS of 5. The rest of the dogs were spread relatively evenly across the remaining groups, with groups 0–3 having 28, 29, 33 and 49 dogs, respectively.

The return to function of dogs per group was 100% in group 5, 99% in group 4, 100% in group 3, 96% in group 2, 86% in group 1 and 64% in group 0 (Table 2). In total, 15 dogs died or were euthanised, as a direct result of complications arising from thoraco-lumbar disc disease and the spinal decompression. This gave a mortality rate of 6% across all groups. Across all the groups, 11 dogs were euthanised because of no improvement in neurological function. Of these 11 dogs, 5 had a pre- and post-operative MFS of 0 and were euthanised at 3 weeks post-surgery as they failed to improve from an MFS of 0. There were three dogs that had a pre-surgical MFS of 1 and a post-MFS of 0, which failed to show any improvement in the MFS; two of these dogs were euthanised 3 weeks after surgery and one was euthanised 2 weeks after surgery at the owner’s request. There were two dogs with a preoperative MFS of 2 and a post-operative MFS of 0; these dogs were euthanised at 3 and 4 weeks, respectively, because of a failure to improve from an MFS of 0. There was one dog with a preoperative MFS of 4 and a post-operative MFS of 0, and this dog was euthanised at 10 days because of severe self-trauma to the hind limb and penis. Four dogs died because of complications that arose as a direct result of thoraco-lumbar disc disease or the decompressive surgery. Of these four dogs, two dogs developed progressive ascending or descending myelomalacia within 72 h of surgery, and the other two died of acute bronchopneumonia from suspected aspiration within 5 days of surgery. Causes of death were confirmed after post-mortem examination of the four dogs (Table 3).

In groups 2–5, the mortality rate was low with three dogs that were euthanised or died. The majority of mortalities were seen in groups 0–1, with 12 dogs dying or being euthanised because of complications that arose directly as a result of intervertebral disc disease or the surgery itself.

During the study, six dogs died from conditions unrelated to thoraco-lumbar disc disease or the decompressive surgery (Table 3).

**TABLE 3:** A comprehensive summary of the minor and major complications seen with mini-hemilaminectomy decompressive spinal surgery.

Modified Frankel Score group	Minor complications			Major complications				Total complications
	Surgical site infection	Urinary tract infection	Seroma	Ascending/descending myelomalacia	Bronchopneumonia	Repeat disc extrusion	No neurological improvement	
5	0	0	1	0	0	1	0	2
4	1	1	0	0	0	0	1	3
3a and b	0	0	0	0	0	0	0	0
2	1	1	0	0	1	0	2	5
1	1	3	1	0	1	0	3	9
0	1	3	0	2	1	0	7	14

Group 5 showed both the shortest mean days to micturition and return to ambulation of 1.5 days of 2 days, respectively. This was followed by group 4, 1.8 days and 2.6 days, respectively; group 3, 4.3 days and 7.6 days, respectively; group 2, 6.4 days and 10.1 days, respectively; group 1, 9.3 days and 16.1 days, respectively; and finally, group 0, 11.9 days and 19.3 days, respectively (Table 2).

Complications (major and minor) were seen in 32 of the surgeries, with a total complication rate of 13% for mini-hemilaminectomy. Group 0 had the highest complication rate with 14 (44%) complications. The least complications were recorded in group 3, with no complications being recorded (Table 3).

A significant (*p* < 0.05) negative correlation (*r* = –0.68) was observed between preoperative MFS and interval to regaining micturition. A significant (*p* < 0.05) negative (*r* = –0.59) correlation was identified between preoperative MFS and interval to regaining ambulation. A significant (*p* < 0.05) negative (*r* = –0.61 and –0.67) correlation was observed between preoperative MFS and development of minor and major complications, respectively.

## Discussion

It is widely accepted that decompressive surgery across all MFS grades of thoraco-lumbar IVDE is an effective treatment in the majority of affected dogs (Langerhuus & Miles 2017). Hemilaminectomy has shown to have higher success rates than a dorsal laminectomy (McKee 1992). This article shows similar outcomes with a mini-hemilaminectomy to a recent similar publication on the outcomes of hemilaminectomy (Skytte & Schmökel 2018). Overall, in this study, 236 (94%) out of 255 dogs that had undergone mini-hemilaminectomy recovered across all the groups (Table 2).

The most common breed represented in this study was the Dachshund, which is well documented in the literature and is in agreement with the current study group (Forterre et al. 2010; Ingram et al. 2013; Skytte & Schmökel 2018). Group 4 (MFS 4) had the highest number of dogs in this study. Group 5 (MFS 5) had the lowest number of dogs in this study. The rest of the groups had a relatively even spread of dogs represented (Table 2).

The majority of dogs (211; 84%) in this study only had a single surgery in the time period examined. Only 35 (14%) dogs had a second repeat surgery in this study. This is higher than that in previous reports of repeat IVDE at another space (Dhupa et al. 1999b). All of the repeat episodes in this study were separate disc extrusions at a different site from the previous surgery. An explanation for this may be that most patients presented are dachshunds (Aikawa et al. 2012) that have been shown to have a higher incidence of disc extrusion. In the study, only five dogs (2%) had a third surgery. The data for the time period of 2012–2014 were examined, and only if a patient had a repeat thoraco-lumbar disc extrusion in this time period, was it recorded. The aim was not to look at the incidence of repeat disc extrusion. It has been included for completeness. A limitation of this study is that none of the dogs were followed for a set time period; only data on the hospital records were examined. This could mean that the incidence of a recurrent disc extrusion may have actually been higher than reported.

Dogs with an MFS of 0 had the lowest recovery rate, with only 19 out of 28 (64%) dogs showing an improvement in neurological function. A preoperative MFS of 0 is associated with the worst possible prognosis in thoraco-lumbar disc extrusion. The reported recovery rates in the literature are around 60% (Kranenburg et al. 2013; Skytte & Schmökel 2018). The use of a mini-hemilaminectomy shows similar results in this study.

Group 0 or dogs with a preoperative MFS of 0 showed a statistically significant, lower recovery rate compared with all the groups (Table 2). In these dogs, only 19 out of 28 dogs (64%) recovered. A preoperative MFS of 0 has shown to have the worst outcomes when treated with a hemilaminectomy, and the outcomes seen in this study in dogs treated with a mini-hemilaminectomy and an MFS of 0 are similar to those seen in dogs treated with a hemilaminectomy as reported in the literature (57% – 61%) (Aikawa et al. 2012; Langerhuus & Miles 2017; McKee 1992). Group 1 dogs with a preoperative MFS of 1 showed a significantly worse recovery rate compared with groups 2–5, but a significantly better recovery rate compared with group 0. These dogs had significant preoperative neurological dysfunction, but 25 of 28 dogs (89%) still recovered after mini-hemilaminectomy. This is comparable with what is reported for hemilaminectomy (93%) (Aikawa et al. 2012; Langerhuus & Miles 2017).

Group 2 or patients with an MFS of 2 had a significantly (*p* < 0.05) improved recovery rate compared with groups 1 and 0. These patients had significant neurological dysfunction prior to surgical decompression. However, 47 out of 49 dogs (96%) showed acceptable return to neurological function (Table 2).

Dogs with an MFS of 3 and 5 had shown recovery of all 33 and 14 dogs, respectively. This is similar to what has been reported in similar studies (Langerhuus & Miles 2017; Levine & Caywood 1984; Skytte & Schmökel 2018). One dog in group 4 did not recover ambulation. This is not unheard of in spinal cord decompressive surgeries, as this was the group with the largest number of patients in the study. The recovery rate was still 98% in this group. It can be assumed that there may be cases in groups 3 and 4, which would not have recovered once the numbers in these groups increase. There is always a risk of not having a full neurological recovery in spinal decompressive surgeries. This needs to be discussed with owners prior to surgery.

This study shows that patients presenting for mini-hemilaminectomy with a preoperative MFS of 5–2 show excellent post-operative recoveries similar to those reported for a hemilaminectomy in the literature (Table 2) (Langerhuus & Miles 2017; Skytte & Schmökel 2018). This makes mini-hemilaminectomy a viable option for decompression of the spinal cord.

The preoperative MFS showed a statistically significant inverse relationship with the length of time to return of micturition. This was in line with similar studies using hemilaminectomy for surgical decompression (Atalan et al. 2002; Skytte & Schmökel 2018). This is suspected to be related to the degree of spinal cord injury from the IVDE as opposed to the selection of mini-hemilaminectomy versus hemilaminectomy.

In this study, dogs with an MFS of 0 showed the highest mean days to micturition of all the groups. This was, however, shorter than seen in a similar study where a hemilaminectomy was performed as the decompressive surgery. That study, however, had much lower number of cases in the groups (Skytte & Schmökel 2018). The trends in this comparative study and our study were, however, similar. Group 5 (MFS 5) showed the shortest mean time to micturition, and this was expected as these patients had the lowest level of neurological dysfunction. However, the fact that the mean time to micturition was more than 1 day shows that there is a degree of neurological dysfunction caused by decompressive surgery, regardless of the technique. This is, however, reversible in most cases (Table 2).

Group 5 had the shortest mean time to ambulation (2 days); unfortunately, the majority of the other studies did not look at the MFS of 5 dogs’ recovery. Our data are comparable with the trends seen in these studies when looking at the MFS of 4 dogs. Dogs in group 4 showed similar mean days to ambulation compared with published studies (Aikawa et al. 2012; Skytte & Schmökel 2018). In this study, dogs with an MFS of 0 had the longest mean days to return of ambulation; however, this was significantly shorter in a comparative study by Skytte et al. As stated before, the numbers of their dogs with an MFS of 0 was significantly lower than our study. The one patient with a prolonged recovery would thus have skewed the mean days because of low case numbers in that group of dogs in that study (type 2 statistical error). Our group had a larger number of patients with a smaller standard deviation. This could indicate that days to ambulation with a hemilaminectomy would approach what was seen in our study with a mini-hemilaminectomy if more cases were included in the study by Skytte et al 2018.

Mini-hemilaminectomy compares favourably with hemilaminectomy for decompression of the spinal cord from acute IVDE in terms of mean time to ambulation, mean time to micturition and recovery rates for the respective neurological groups (Skytte & Schmökel 2018).

Complications were recorded in almost all of the groups of dogs. However, the complication rate for all of the dogs across all the groups having a mini-hemilaminectomy was relatively low. Complications were seen in 32 dogs (13%) of 252 mini-hemilaminectomies. There was a statistically significant inverse relationship between the incidence of complications and the preoperative MFS of the dog.

Group 0 had the highest complication rate, with 14 of 28 dogs (50%) having a complication in the immediate post-operative recovery period. Dogs with an MFS of 0 accounted for 10 out of 28 dogs having major complications that lead to a repeat surgery or death or euthanasia. In dogs where there was no recovery of neurological function, this was considered a major complication leading to negative outcomes. This was the most common reason for negative outcomes seen in 10 dogs. This is similar to the published figures of dogs with an MFS of 0 having a hemilaminectomy of around 36% of dogs not recovering (Aikawa et al. 2012; Langerhuus & Miles 2017; Skytte & Schmökel 2018). Of the 10 dogs, 2 died of ascending or descending myelomalacia, and this is seen in a small percentage of thoraco-lumbar disc extrusions and appears to be related to the degree of spinal trauma. Given that these patients had the worst possible spinal injury, it is expected that the highest number of cases would have had a preoperative MFS of 0. This is more often seen in the literature on dogs with an MFS of 0 and with an incidence of 14%, but can occur in any patient with IVDE with an overall incidence of around 2% across all MFSs (Balducci et al. 2017; Baird et al. 2014; Forterre et al. 2010). Of the 10 dogs, 1 died of bronchopneumonia, and this was confirmed on post-mortem examination. This has been observed in patients having spinal cord decompression and is a known complication; a number of theories have been put forward such as bacterial implantation in the pleura during the surgical approach to the spinal column or from aspiration of the regurgitate gastric content under general anaesthesia (Java et al. 2009).

Only four minor complications were seen in group 0, with three of these complications being urinary tract infections. In this study, dogs were expressed in most cases. A urinary catheter was only used if expression was not possible. The need for catheterisation was not recorded but did not happen often based on clinician experience. This is often seen in dogs that have IVDE because of the retention of urine in the post-operative recovery. This residual amount of urine will predispose them to urinary tract infections (Atalan et al. 2002; Bubenik et al. 2007). Dogs in group 0 had the longest mean days to return of micturition. This would make urinary tract infections more common, given that there is more time for the residual urine, retention predisposes to the development of an infection (Bubenik et al. 2007). With expression of the bladder being the main method of voiding in this study post-surgery the flushing effect of the urine would likely prevent an ascending urinary tract infection. It would have been ideal to look at the proportion of female dogs that developed an infection compared with male dogs in this study. This would evaluate the effect of urethral length on prevention of an ascending urinary tract infection. This was not performed in this study.

Group 1 had the second highest complication rate of nine complications out of 29 dogs (31%), which was statistically more than groups 2–5 but statistically less than group 5. There were four major complications seen in group 1, the majority of these, three dogs, failed to show improvement in neurological function and were euthanised. In group 1, one dog died of bronchopneumonia, which was confirmed on post-mortem examination. This could have been from a subclinical issue arising under anaesthesia as discussed for bronchopneumonia in group 0. Group 1 had the highest number of minor complications, with three of these dogs having urinary tract infections. Once again, these patients had the second longest time to micturition and ambulation, so urine retention would have been observed in these dogs for longer time than that in groups 2–5. This would explain why the incidence of urinary tract infections was highest in groups 0 and 1. There was one surgical site infection in group 1; this together with the surgical site infection in group 0 and groups 2 and 4 gives a surgical site infection rate of 4 dogs out of 252 (1.6%). This is significantly higher compared with what was published for hemilaminectomy (0.6%), but still below the published infection rate in clean procedures in veterinary medicine (2% – 4%) (Dyall & Schmökel 2018). Our rate of urinary tract infection was 7 dogs out of 252 (3%); this is significantly less than reported (27%) (Bubenik et al. 2007). A culture was only taken in our study if the patient showed signs of haematuria, stranguria or dysuria. This may have led to a false low number of dogs with bacteriuria. None of our patients developed issues related to an undiagnosed urinary tract infection.

There were only two major complications seen in groups 3–5, the most devastating one was seen in group 4 where a patient was euthanised because of failure to show neurological improvement post-surgery. This patient had a preoperative MFS of 4 and developed a post-operative MFS of 0. The owners declined further diagnostic imaging or a post-mortem examination after euthanasia. There were still 98 patients out of 99 (99%) that recovered in group 4, with a similar recovery rate to what is reported for a hemilaminectomy. The other major complication was observed in group 5 and was a repeat thoraco-lumbar disc extrusion at another disc space. This was treated as a separate procedure, and the dog made a delayed but full recovery.

The other minor complications seen were three incidences of seromas, but these did not affect the improvement of the patient or delay discharge from the hospital. This figure may be underestimated as patients doing well could have had the sutures removed by another veterinarian and the true incidence of seroma formation not reported. The seromas reported were only in patients during the post-operative hospitalisation period.

## Conclusion

A mini-hemilaminectomy gives a similar recovery in terms of mean days to return of micturition, ambulation and complication rates to that published for hemilaminectomy.
